# Noninvasive Real-Time Mortality Prediction in Intensive Care Units Based on Gradient Boosting Method: Model Development and Validation Study

**DOI:** 10.2196/23888

**Published:** 2021-03-25

**Authors:** Huizhen Jiang, Longxiang Su, Hao Wang, Dongkai Li, Congpu Zhao, Na Hong, Yun Long, Weiguo Zhu

**Affiliations:** 1 Department of Information Center, State Key Laboratory of Complex Severe and Rare Diseases Peking Union Medical College Hospital Chinese Academy of Medical Science and Peking Union Medical College Beijing China; 2 Department of Critical Care Medicine, State Key Laboratory of Complex Severe and Rare Diseases Peking Union Medical College Hospital Chinese Academy of Medical Science and Peking Union Medical College Beijing China; 3 Digital Health China Technologies Co., Ltd Beijing China

**Keywords:** real time, mortality prediction, intensive care unit, noninvasive

## Abstract

**Background:**

Monitoring critically ill patients in intensive care units (ICUs) in real time is vitally important. Although scoring systems are most often used in risk prediction of mortality, they are usually not highly precise, and the clinical data are often simply weighted. This method is inefficient and time-consuming in the clinical setting.

**Objective:**

The objective of this study was to integrate all medical data and noninvasively predict the real-time mortality of ICU patients using a gradient boosting method. Specifically, our goal was to predict mortality using a noninvasive method to minimize the discomfort to patients.

**Methods:**

In this study, we established five models to predict mortality in real time based on different features. According to the monitoring, laboratory, and scoring data, we constructed the feature engineering. The five real-time mortality prediction models were RMM (based on monitoring features), RMA (based on monitoring features and the Acute Physiology and Chronic Health Evaluation [APACHE]), RMS (based on monitoring features and Sequential Organ Failure Assessment [SOFA]), RMML (based on monitoring and laboratory features), and RM (based on all monitoring, laboratory, and scoring features). All models were built using LightGBM and tested with XGBoost. We then compared the performance of all models, with particular focus on the noninvasive method, the RMM model.

**Results:**

After extensive experiments, the area under the curve of the RMM model was 0.8264, which was superior to that of the RMA and RMS models. Therefore, predicting mortality using the noninvasive method was both efficient and practical, as it eliminated the need for extra physical interventions on patients, such as the drawing of blood. In addition, we explored the top nine features relevant to real-time mortality prediction: invasive mean blood pressure, heart rate, invasive systolic blood pressure, oxygen concentration, oxygen saturation, balance of input and output, total input, invasive diastolic blood pressure, and noninvasive mean blood pressure. These nine features should be given more focus in routine clinical practice.

**Conclusions:**

The results of this study may be helpful in real-time mortality prediction in patients in the ICU, especially the noninvasive method. It is efficient and favorable to patients, which offers a strong practical significance.

## Introduction

Patients in intensive care units (ICUs) are usually suffering from the most severe and complicated diseases. Thus, they require more intensive care and hospital resources [1]. Research shows that the cost of ICUs accounts for 22% of total hospital costs [2]. The cost of doctors and nurses in the ICU is also a massive burden. Therefore, hospitals usually use scoring systems to help assess patients’ risks and then place more efforts on improving the patient care and management. Scoring systems such as the Acute Physiology and Chronic Health Evaluation (APACHE) [3] systems II, III, and IV; the Simplified Acute Physiology Score II (SAPS II) [4]; and the Sequential Organ Failure Assessment (SOFA) score [5] are commonly used to estimate the illness severity of patients in the ICU [6,7]. However, the scoring systems cannot reflect the condition of patients in real time, and clinical staff must spend plenty of time calculating the scores to make decisions. Further, the scores alone are insufficient for the needs of the clinical staff. Johnson and Mark [8] found that the gradient boosting method outperformed the scoring systems on predicting mortality, which provided inspiration for our study.

Meanwhile, the severity and mortality of ICU patients can be specifically assessed in real time using machine learning methods. This would allow doctors and nurses to prepare lifesaving interventions ahead of time and provide families with more time to make decisions [9]. Hence, precisely predicting the mortality of ICU patients is significant. Machine learning technology has significantly changed lives in many aspects in recent years, even in the health care field [10,11]. Usually, the shortest time period for predicting mortality is 24 h [12,13], which is not sufficient for the ICU staff to obtain the real-time condition of patients. Kim et al [14] presented a deep learning method to predict the mortality of patients 6 h to 60 h prior to death, where the time period was a little longer than the real time. With regard to machine learning techniques, the ensemble and neural network models demonstrate better performance in predicting mortality [2]. Brand et al [15] proposed a deep learning method to predict mortality based only on heart rate, respiratory rate, and blood pressure, which had an accuracy of 76.3%, but its performance was not as good as that of other methods. Besides, the neural network model cannot interpret the gap between the input and the output. Further, it is vulnerable to attack when the training set is inadvertently being modified [16].

In this study, we established a real-time mortality prediction model based on clinical data where we explored a noninvasive method to predict mortality by only monitoring features. Because frequent laboratory examination can cause physical trauma to patients whose bodies are already weak, using a model that can show general performance and is noninvasive is clinically meaningful.

## Methods

### Data Sources

We used the ICU data from Peking Union Medical College Hospital from 2013 to 2018. A total of 13,649 patients were investigated in our experiments with the privacy information filtered out. We mined features from three types of data: real-time monitoring, laboratory, and scoring data. The main features from the monitoring data are listed in Multimedia Appendix 1.

### Prediction Model

**Figure 1:**
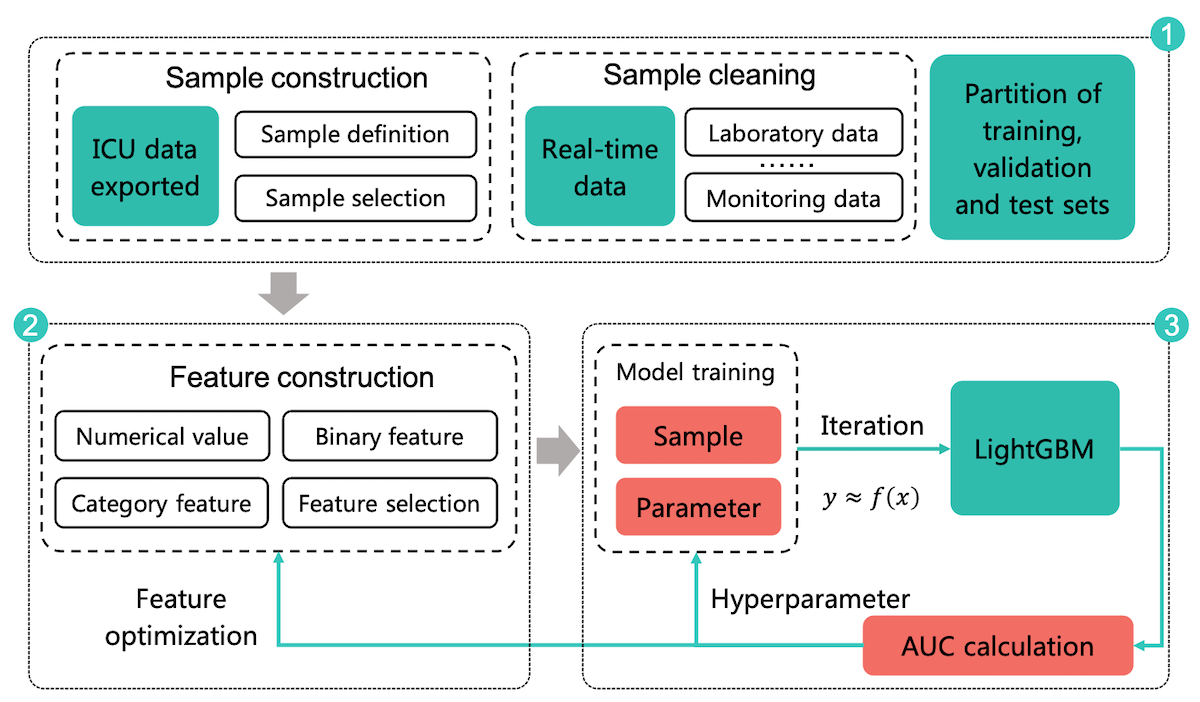
Modeling process, including sample construction and cleaning, feature engineering, model training, and optimization. AUC: area under the curve; ICU: intensive care unit.

In this study, we constructed the real-time mortality prediction model based on the clinical data. The data of the patients in the ICU were updated all the time, and the model could predict each patient’s mortality once the data were updated; the model could predict the mortality after 2 h at any time if the data were not updated during the 2 h period. Therefore, it is a real-time prediction model. The modeling process involved three steps, which are shown in Figure 1. First, we constructed and cleaned the sample data according to the clinical data. Second, after dividing the data into training and test sets, we normalized all types of data as features. Third, we used the LightGBM method to train the data and optimized the model by adjusting the parameters. LightGBM and XGBoost are both gradient boosting decision tree methods, and LightGBM has good performance and high training efficiency [17]. In this paper, we also compared the performance of the LightGBM and XGBoost methods.

### Sample Construction

Usually, patients in ICUs are weak and at high risk. Therefore, focusing on the real-time condition of an ICU patient by the clinical staff is meaningful. In this research, we predicted the mortality of a patient after 2 h based on the clinical data.

**Figure 2:**
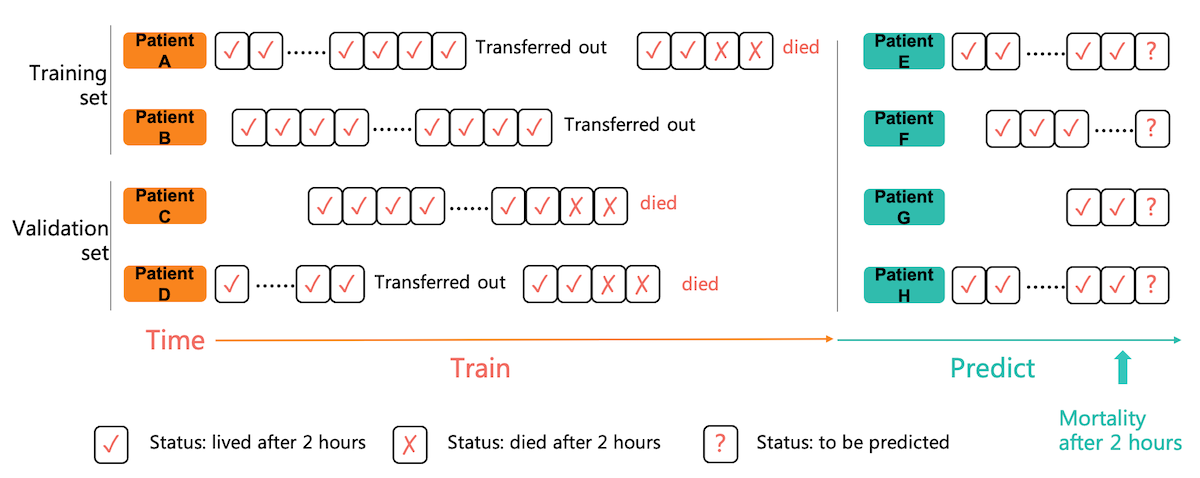
Feature engineering process. Each square represents a 1 h record in the intensive care unit (ie, one sample). The symbols in the squares indicate the status of the patient.

Figure 2 shows how we constructed the data by the hour. One record of a patient was captured in each hour, and each patient may have a sample sequence based on the timeline. As a result, there might be several samples for one patient. For example, patient A had been admitted to the ICU twice, and patients B and C had each been admitted to the ICU once. There were 2 samples for patient A. During patient A’s second stay in the ICU, the third box contained a cross, representing a status of “died after 2 h,” so he/she died 2 h after he/she was admitted into the ICU because one box meant one sample in 1 h. The data of patients A and B were the training data, and the data of patients C and D were the validation data. Samples of patients E, F, G, and H were the test data set. Then, the samples were constructed according to the process shown in Figure 2. For the 13,649 patients, we constructed 1,172,652 samples in all.

### Feature Engineering

Feature engineering is the key process in machine learning. The modeling performance depends on the feature engineering quality to a large extent.

In this study, two data types existed: numerical and categorical data from the monitoring, laboratory, and scoring data. For the numerical data, we directly considered the numerical value as the feature, such as the heart rate and temperature. The categorical data included gender and positive or negative status. We used the LabelEncoder method [18] to normalize these categorical data. LabelEncoder is a method that converts text data into multinumeric values. It can convert two-class and multiclass features. For example, the positive and negative states were represented by 0 and 1, respectively. We left the missing value blank to ensure the authenticity of the data.

### Model Training

In this research, we needed to predict the real-time condition of a patient 2 h after each moment. Actually, this process was a binary classification problem (ie, life or death). LightGBM is a gradient boosting method that is superior in dealing with the binary classification problem and has high efficiency and performance, especially in dealing with structured data. The 1,172,652 samples were randomly divided into three parts. One-third of the samples was set as the training set, one-third was set as the validation set, and the rest was set as the test set. The area under the curve (AUC) was used to evaluate the model’s performance.

Based on different features, we constructed five real-time mortality prediction models:

- RMM: based on monitoring features;
- RMA: based on monitoring features and APACHE;
- RMS: based on monitoring features and SOFA;
- RMML: based on monitoring and laboratory features; and
- RM: based on all monitoring, laboratory, and scoring features.

## Results

**Figure 3:**
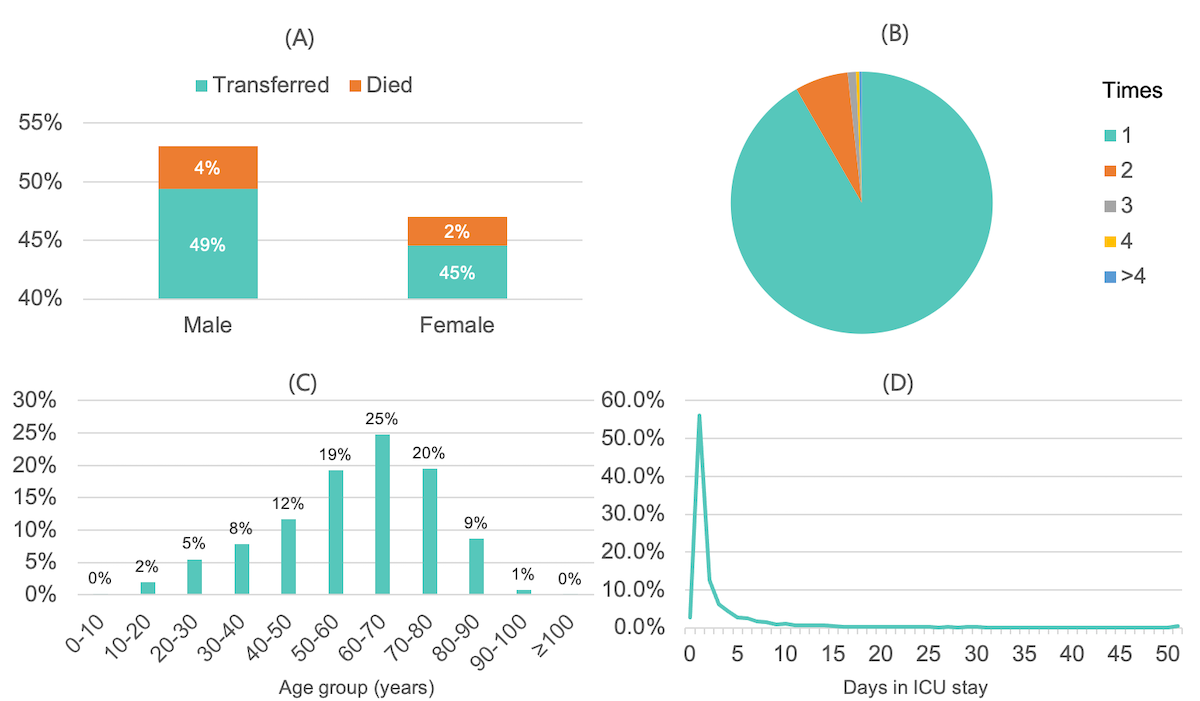
Distribution of the data set. (A) Proportion of patients that were transferred out of the intensive care unit (ICU) or died, according to gender. (B) Distribution of the number of times patients transferred into the ICU. (C) Age group distribution of ICU patients. (D) Length of stay of patients in the ICU.

In presenting the results of our study, we will focus on the results of the models in the test set. Figure 3 shows the distributions and proportions of patients in the ICU in the data set. Figure 3A shows that male patients in the ICU outnumbered female patients irrespective of whether they were transferred out or died. Figure 3B shows that more than 12,510 patients were transferred into the ICU only once, and 898 patients stayed in the ICU twice. Patients between the ages of 50 and 80 years accounted for 8700 of the total number of patients, as shown in Figure 3C. In addition, patients between the ages of 60 and 70 years represented the largest group, accounting for one-quarter of the total number of patients. Figure 3D shows the length of stay of patients in the ICU in a single visit; we can observe that most patients stayed in the ICU for fewer than 5 days.

**Figure 4:**
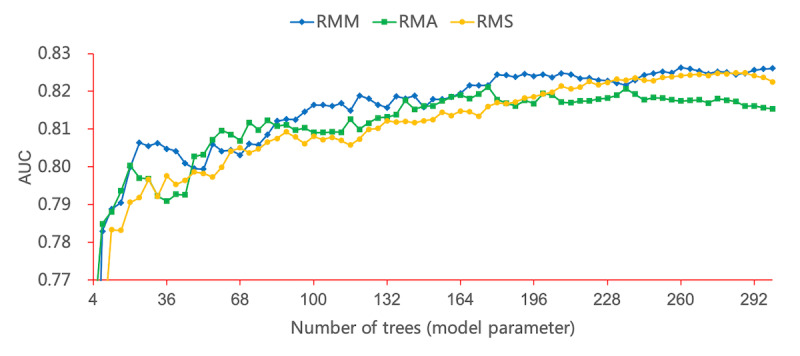
Performance of the RMM, RMA, and RMS models with parameter variation. Each point on the line represents one experiment. AUC: area under the curve.

**Figure 5:**
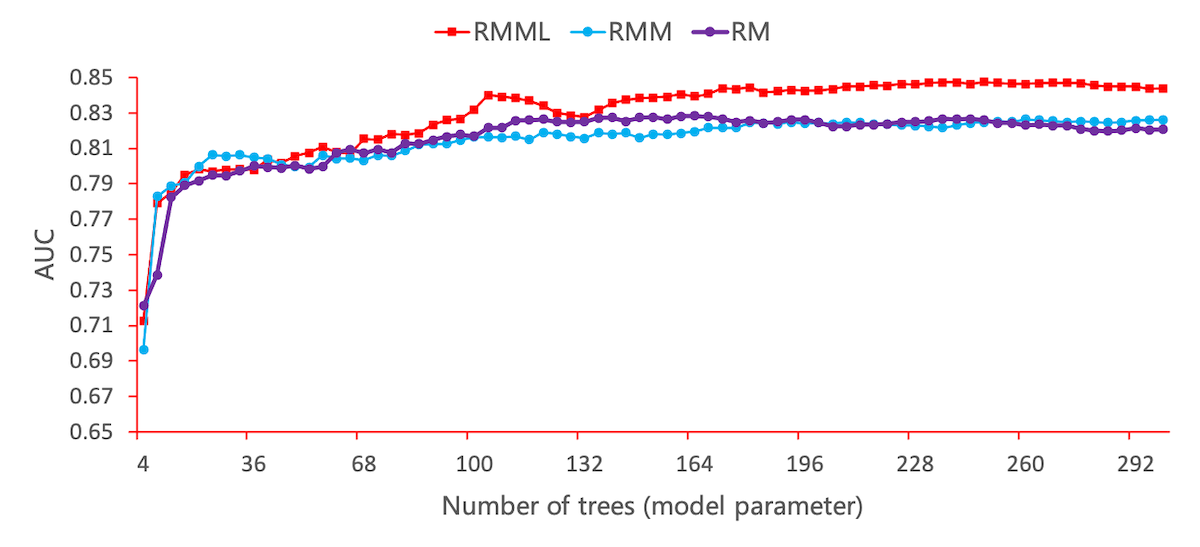
Performance of the RMML, RMM, and RM models with parameter variation. Each point on the line represents one experiment. AUC: area under the curve.

First, we evaluated the influence of the scoring systems through extensive experiments; the results are shown in Figures 4 and 5. Figure 4 shows that the RMM model outperformed the RMA and RMS models. Overall, all three models showed an upward trend with the increase in tree number and became stable after the tree number reached 200. The RMS model demonstrated better performance than the RMA model. Therefore, the SOFA scoring system was more valuable than the APACHE scoring system in predicting mortality. Compared with the RMA and RMS models, the RMM model was superior and demonstrated the best performance (AUC 0.8264) when the tree number was 299.

Figure 5 shows the results of the experiments that we conducted on the RMML, RMM, and RM models to compare their performance in terms of the monitoring, laboratory, and scoring features. The RMML model exhibited the best performance based on the monitoring and laboratory features than the other two models. When the tree number was 234, RMML obtained the best AUC (0.8476). In the RM model, monitoring, laboratory, and all scoring features were considered. The RM model exhibited worse performance than the RMM model.

In addition, we repeated the experiments above using XGBoost. The AUCs of XGBoost were relatively lower than those of LightGBM, as shown in Table 1. The best performance with XGBoost was 0.8452 on the RMML model and 0.8154 on the RMM model. As well, we showed the RMML and RMM models using LightGBM and XGBoost on the validation set, and the results are shown in Table 1. Therefore, LightGBM outperformed XGBoost in these experiments.

**Table 1:** Performance of the RMML and RMM models using LightGBM and XGBoost.

		Area under the curve	
Model and method		Test set	Validation set
**RMML**			
	LightGBM	0.8476	0.8483
	XGBoost	0.8452	0.8466
**RMM**			
	LightGBM	0.8264	0.8269
	XGBoost	0.8154	0.8167

**Figure 6:**
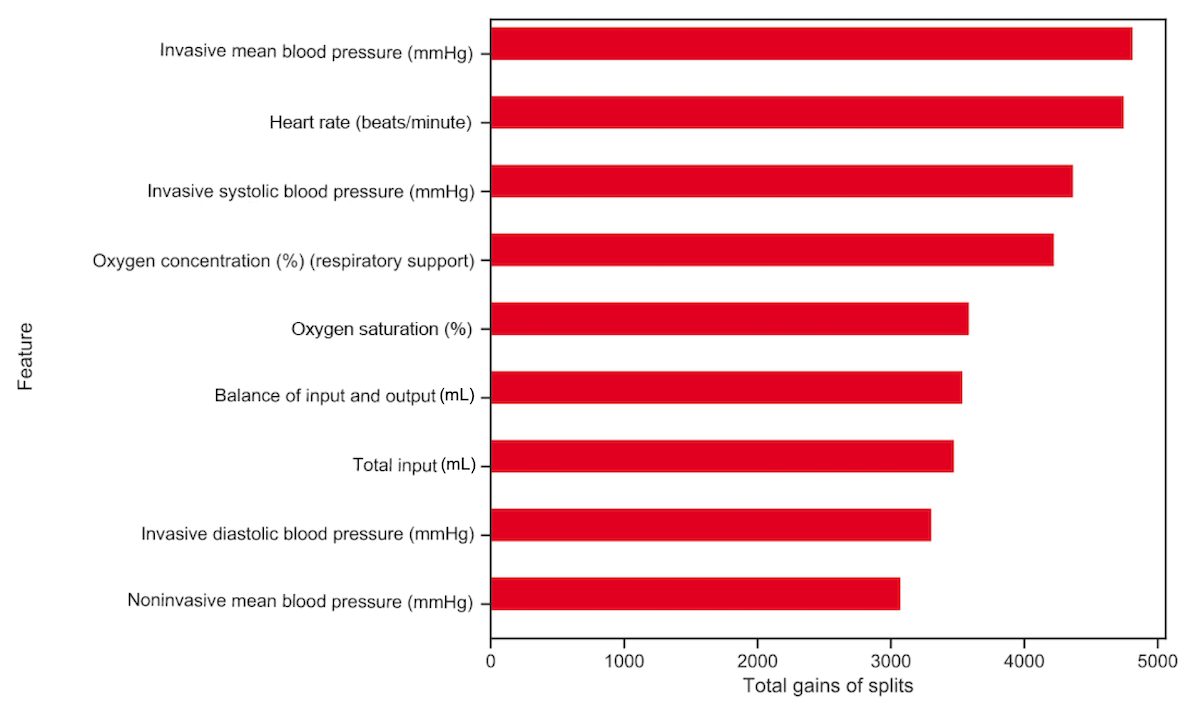
Top nine features relevant to mortality prediction. The horizontal bar represents the gain of each feature in the model; a bigger gain means more relevance and importance in the mortality prediction.

Because the RMML model demonstrated the best performance, we analyzed the relevant features that predicted mortality in that model. Figure 6 shows the top nine features relevant to the mortality prediction. The “gain” of the feature splitting implies the importance of the feature in the model, which was computed during the model training. Thus, the bigger the gains of the feature, the more important the feature was in the model. It was shown that invasive mean blood pressure was the most important feature related to mortality prediction. Among the top nine features, heart rate, invasive systolic blood pressure, oxygen concentration, oxygen saturation, balance of input and output, total input, invasive diastolic blood pressure, and noninvasive mean blood pressure were all vital sign features in the monitoring. “Balance of input and output” was the difference between input and output data, while “total input” was the input data only. They all demonstrated a relatively strong correlation with the mortality prediction.

**Figure 7:**
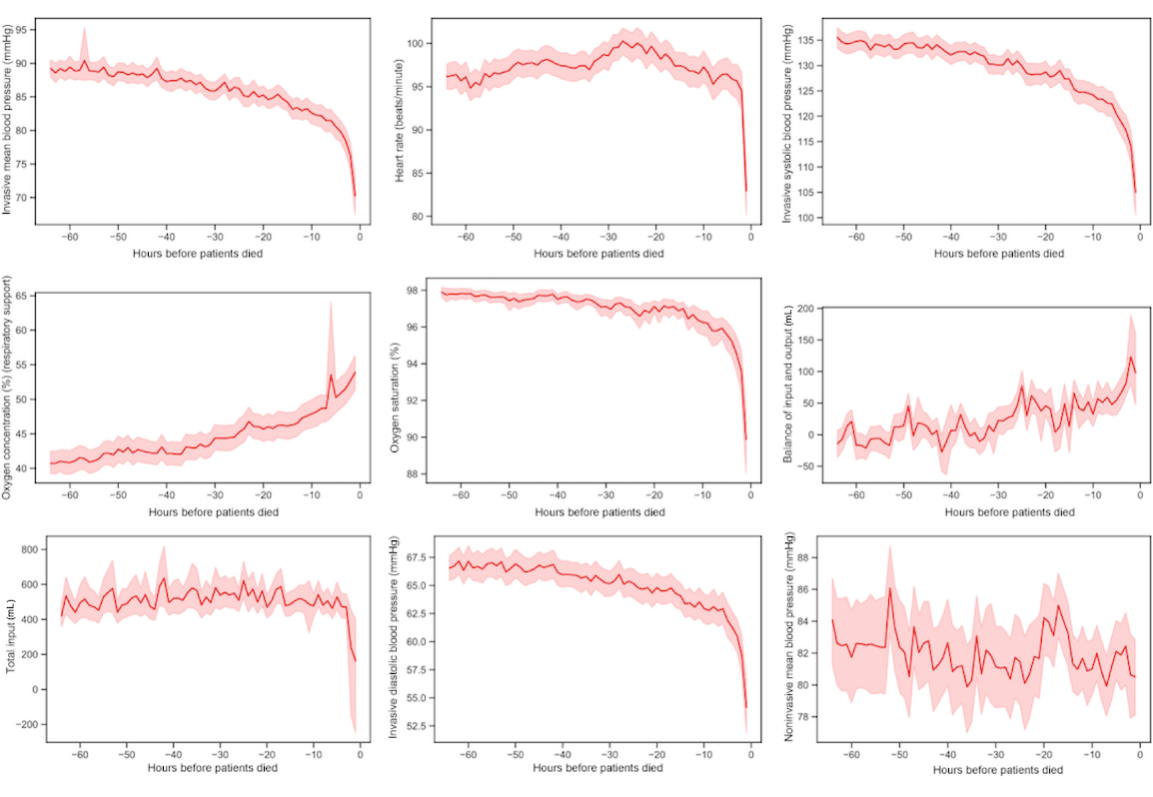
Variation in the top nine features relevant to mortality prediction with time. The abscissa represents 64 h before the patients died, and the ordinate represents the value of the feature.

In addition, we exploited the variation in each of the top nine features with time during the last 64 h before a patient died. Figure 7 shows that all nine features showed an obvious trend with the time variation. “Oxygen concentration” and “balance of input and output” exhibited an upward trend during the final 64 h before the patient died. The other seven features all decreased with time during the final 64 h before the patient died. For example, invasive diastolic blood pressure exhibited a downward trend and sharply declined in the last 5 h. Similarly, the top eight features all rapidly changed in the last 5 h. The “noninvasive mean blood pressure” exhibited dithering but an overall decreasing trend.

## Discussion

In this paper, we used clinical data to predict the real-time mortality of ICU patients. Several models were established based on different features. Extensive experiments showed that the models that used the machine learning method were superior to the scoring systems. More importantly, they can be employed to predict real-time mortality in a noninvasive manner.

Constant care of ICU patients is necessary against their life-threatening conditions. Intensive care is based on more financial support and more professional hospital staff [19,20]. The US health care spending was approximately 17% of the gross domestic product (GDP) in 2011 and may reach 26% of the GDP by 2035 [21]. In addition to the cost, the mortality rate in ICUs cannot be ignored. Studies show that ICUs have the highest mortality rate of all hospital units (16.2% [22] and 22.4% [23]). Therefore, helping predict patient mortality in ICUs is significant, as it could save time for nurses and doctors by more efficiently measuring the risk of ICU patients. It would be better if there was less trauma to patients in the clinical process.

Commonly, hospital staff use scoring systems to help predict the severity status of ICU patients. Most of these scoring systems calculate the scores based on the worst values during the first 24 h after ICU admission [24]. The SAPS score only uses the data in the first hour after ICU admission, which are more robust because the missing data have a lesser effect on specificity [25]. Saleh et al [24] compared APACHE II and III, SAPS II, and SOFA and showed that APACHE II and III demonstrated better performance than the others. However, Yap et al [26] verified that the National Early Warning Score demonstrated the best performance for predicting the severity status of patients with emphysematous pyelonephritis patients. Tan et al [27] explored the ability of the scoring systems to predict sepsis mortality in the short term (less than 30 days in the hospital) and long term (more than 30 days). They discovered that the sensitivity and specificity were similar in both factors, whereas geographical region had a significant effect on the short-term mortality prediction. Therefore, the scoring systems can show different performance on different diseases and under different situations [28,29]. In addition, Nielsen et al [30] compared the APACHE II and SAPS II with the aggregation of the APACHE II and SAPS II, and the aggregation of APACHE II and SAPS II outperformed each single model. Similarly, Fei et al [31] presented the use of the fibrin degradation product level and APACHE II scores in parallel to improve the prediction performance.

Machine learning technologies have been increasingly used in the health care field because of their excellent performance [20,32]. Further, machine learning models have been confirmed to perform better in predicting the severity status of ICU patients than the scoring systems. Henry et al [33] proposed a supervised learning model to predict the risk of patients getting septic shock, and machine learning was found to have higher sensitivity and specificity than the scoring systems. An ensemble machine learning model was investigated by Pirracchio et al [2], and the results showed better performance for the machine learning model than for the common scoring systems. In recent years, many studies have focused on using the neural network model to predict mortality [34,35]. Most of the experiments demonstrated that the neural network model outperformed the other models. Norrie [36] innovatively proposed a prespecified library of models and established an optimum model. However, the neural network model is difficult to explain in terms of the black box principle [32,37], which is not clear for high recursion [38]. Using the inherently interpretable models is vital and important in health care because decisions in health care involve high stakes [39]. Awad et al [20] demonstrated that the decision tree model is interpretable and better than the neural network model in predicting mortality. Similarly, Blanco-Justicia et al [40] used a depth-limited decision tree model to avoid the black box problem. In reality, the gradient boosting methods usually perform better than the deep learning method on structured data, especially on a small data set.

The limitation of this study is that the data were obtained from one hospital only. The structure and quality of data may vary in different hospitals. In the future, we would try to improve our model based on multicenter data.

In the present study, we constructed the real-time mortality prediction model based on the monitoring, laboratory, and scoring data. Compared with the RMM, RMA, RMS, and RM models, the RMML model demonstrated the best performance. Moreover, we found that the invasive mean blood pressure, heart rate, and invasive systolic blood pressure were the top three features relevant to the mortality prediction. In addition, the RMM model performed better than the RMA and RMS models. Therefore, noninvasively predicting real-time mortality would be meaningful. Not only can the results of our research provide support for decision making by clinical staff, but our method is also better for patients because the real-time mortality prediction is noninvasive.

## Appendix

Multimedia Appendix 1 The main monitoring features.

## Abbreviations

APACHE: Acute Physiology and Chronic Health Evaluation
AUC: area under the curve
GDP: gross domestic product
ICU: intensive care unit
SAPS II: Simplified Acute Physiology Score II
SOFA: Sequential Organ Failure Assessment
