# Supplementary material for: Noninvasive Real-Time Mortality Prediction in Intensive Care Units Based on Gradient Boosting Method: Model Development and Validation Study
Source: JMIR Med Inform. 2021 Mar 25;9(3):e23888. doi: 10.2196/23888 (PMC8077746; doi:10.2196/23888)
Supplement: Multimedia Appendix 1 [file medinform_v9i3e23888_app1.docx]

**Multimedia Appendix 1**

The main monitoring features

| 1 | invasive mean blood pressure |
| --- | --- |
| 2 | heart rate |
| 3 | invasive systolic blood pressure |
| 4 | oxygen concentration |
| 5 | SPO2 |
| 6 | balance of input and output |
| 7 | total input |
| 8 | invasive diastolic blood pressure |
| 9 | noninvasive mean blood pressure |
| 10 | total output |
| 11 | temperature |
| 12 | noninvasive systolic blood pressure |
| 13 | noninvasive diastolic blood pressure |
| 14 | ECMO blood flow rate |
| 15 | Transmembrane Pressure |
| 16 | age |
| 17 | Pmean(cmH2O) |
| 18 | respiratory ratio (setting) |
| 19 | inhaled tidal volume |
| 20 | breathing mode |
| 21 | respiratory rate (respiratory support) |
| 22 | patient dehydration |
| 23 | expiratory tidal volume |
| 24 | height |
| 25 | PC(cmH2O) |
| 26 | blood temperature |
| 27 | rectal temperature |
| 28 | replacement fluid |
| 29 | weight |
| 30 | venous pressure |
| 31 | pre diluent |
| 32 | Ppeak |
| 33 | front pump of blood pump |
| 34 | tidal volume |
| 35 | effluent liquor (setting) |
| 36 | respiratory rate (setting)(respiratory support) |
| 37 | post diluent |
| 38 | PS(cmH2O) |
| 39 | effluent liquor |
| 40 | respiratory rate (invasive)(respiratory support) |
| 41 | respiration rate |
| 42 | post diluent (setting) |
| 43 | Pflat |
| 44 | positive end expiratory pressure |
| 45 | arterial pressure |
| 46 | pre diluent (setting) |
| 47 | speed of ECMO |
| 48 | oxygen flow |
| 49 | ultrafiltration rate (setting) |
| 50 | 5%NaHCO3 |
| 51 | gas oxygen concentration of ECMO |
| 52 | blood pump speed |
| 53 | respiratory rate (invasive)(setting)(respiratory support) |
| 54 | central venous pressure |
| 55 | replacement fluid (setting) |
| 56 | blood purification balance |
| 57 | positive end expiratory pressure (setting) |
| 58 | blood purification mode |
| 59 | ultrafiltration rate |
| 60 | gender |
| 61 | respiratory ratio |
| 62 | patient dehydration (setting) |
| 63 | dialysate |
| 64 | oxygen flow of ECMO |
| 65 | flow of ECMO |
| 66 | equipment type (hemofiltration) |
| 67 | ECMO mode |
| 68 | V femoral artery |
| 69 | A femoral artery |
| 70 | V femoral vein |
| 71 | V internal jugular vein |
| 72 | ECMO catheter |
